# Neuropeptide S-initiated sequential cascade mediated by OX_1_, NK_1_, mGlu_5_ and CB_1_ receptors: a pivotal role in stress-induced analgesia

**DOI:** 10.1186/s12929-019-0590-1

**Published:** 2020-01-09

**Authors:** Ming Tatt Lee, Yu-Ting Chiu, Yu-Chun Chiu, Chia Chun Hor, Hsin-Jung Lee, Remo Guerrini, Girolamo Calo, Lih-Chu Chiou

**Affiliations:** 1 Graduate Institute of Brain and Mind Sciences, College of Medicine, National Taiwan University, Taipei, 10051 Taiwan; 2 Graduate Institute of Pharmacology, College of Medicine, National Taiwan University, Taipei, 10051 Taiwan; 3 Faculty of Pharmaceutical Sciences, UCSI University, 56000 Kuala Lumpur, Malaysia; 4 Department of Pharmacology, College of Medicine, National Taiwan University, Taipei, 10051 Taiwan; 5 Department of Chemical and Pharmaceutical Sciences, Laboratorio per le Tecnologie delle Terapie Avanzate (LTTA), Ferrara, Italy; 6 Department of Medical Sciences and National Institute of Neurosciences, Section of Pharmacology, University of Ferrara, 44121 Ferrara, Italy; 7 Graduate Institute of Acupuncture Science, China Medical University, Taichung, 40402 Taiwan

**Keywords:** Neuropeptide S, Orexin, Substance P, Metabotropic glutamate receptor, Endocannabinoid, Periaqueductal gray

## Abstract

**Background:**

Stress-induced analgesia (SIA) is an evolutionarily conserved phenomenon during stress. Neuropeptide S (NPS), orexins, substance P, glutamate and endocannabinoids are known to be involved in stress and/or SIA, however their causal links remain unclear. Here, we reveal an unprecedented sequential cascade involving these mediators in the lateral hypothalamus (LH) and ventrolateral periaqueductal gray (vlPAG) using a restraint stress-induced SIA model.

**Methods:**

Male C57BL/6 mice of 8–12 week-old were subjected to intra-cerebroventricular (*i.c.v.*) and/or intra-vlPAG (*i.pag.*) microinjection of NPS, orexin-A or substance P alone or in combination with selective antagonists of NPS receptors (NPSRs), OX_1_ receptors (OX_1_Rs), NK_1_ receptors (NK_1_Rs), mGlu_5_ receptors (mGlu_5_Rs) and CB_1_ receptors (CB_1_Rs), respectively. Antinociceptive effects of these mediators were evaluated via the hot-plate test. SIA in mice was induced by a 30-min restraint stress. NPS levels in the LH and substance P levels in vlPAG homogenates were compared in restrained and unrestrained mice.

**Results:**

NPS (*i.c.v.,* but not *i.pag.*) induced antinociception. This effect was prevented by *i.c.v.* blockade of NPSRs. Substance P (*i.pag.*) and orexin-A (*i.pag.*) also induced antinociception. Substance P (*i.pag.*)-induced antinociception was prevented by *i.pag.* Blockade of NK_1_Rs, mGlu_5_Rs or CB_1_Rs. Orexin-A (*i.pag.*)-induced antinociception has been shown previously to be prevented by *i.pag.* blockade of OX_1_Rs or CB_1_Rs, and here was prevented by NK_1_R or mGlu_5_R antagonist (*i.pag.*). NPS (*i.c.v.*)-induced antinociception was prevented by *i.pag.* blockade of OX_1_Rs, NK_1_Rs, mGlu_5_Rs or CB_1_Rs. SIA has been previously shown to be prevented by *i.pag.* blockade of OX_1_Rs or CB_1_Rs. Here, we found that SIA was also prevented by *i.c.v.* blockade of NPSRs or *i.pag.* blockade of NK_1_Rs or mGlu_5_Rs. Restrained mice had higher levels of NPS in the LH and substance P in the vlPAG than unrestrained mice.

**Conclusions:**

These results suggest that, during stress, NPS is released and activates LH orexin neurons via NPSRs, releasing orexins in the vlPAG. Orexins then activate OX_1_Rs on substance P-containing neurons in the vlPAG to release substance P that subsequently. Activates NK_1_Rs on glutamatergic neurons to release glutamate. Glutamate then activates perisynaptic mGlu_5_Rs to initiate the endocannabinoid retrograde inhibition of GABAergic transmission in the vlPAG, leading to analgesia.

## Background

Stress-induced analgesia (SIA) is an evolutionarily protective system in mammals for coping with environmental stressors [1]. Several neuropeptides released during stress, such as orexins [2, 3], neuropeptide S (NPS) [4] and substance P [5], may contribute to SIA. However, how these neuropeptide-mediated signals interact to elicit SIA remains unknown.

Orexins, consisting of orexin-A and orexin-B [6], also known as hypocretin-1 and hypocretin-2 [7], are processed from preprohypocretin in hypothalamic neurons in the perifornical area (PFA), lateral hypothalamus (LH) and dorsomedial hypothalamus (DMH) [6, 7]. Orexin receptors, OX_1_ receptors (OX_1_Rs) and OX_2_ receptors (OX_2_Rs), belong to the G-protein coupled receptor (GPCR) family [8]. In addition to being involved in arousal and reward regulation [9], orexins are antinociceptive [10–12] and are involved in SIA [2, 3, 12, 13]. Previously, we have shown that orexins can be released during stress and contribute to SIA, at least in part, via opioid-independent and endocannabinoid (eCB)-dependent signaling [11, 12] in the ventrolateral periaqueductal gray (vlPAG), a crucial midbrain region for the initiation of descending pain inhibition [14, 15]. Specifically, orexins are released during stress [12], and orexins are known to induce antinociception by activating postsynaptic OX_1_Rs to generate 2-arachidonoylglycerol (2-AG) [16, 17], an eCB, through a Gq protein-coupled enzymatic cascade mediated by phospholipase C (PLC) and diacylglycerol lipase (DAGL) [18], culminating in retrograde inhibition of GABA release (disinhibition) in the vlPAG [11, 12].

NPS is an icosapeptide named due to its conserved N-terminal residue, serine, in all species [4]. Central administration of NPS (intra-cerebroventricular, *i.c.v.*) is antinociceptive [19–21]. The site of this antinociceptive action could be the PAG, where the mRNA transcript of NPS receptors (NPSRs) is abundant [22, 23], or other NPSR-rich brain regions, such as the amygdala and hypothalamus [22]. All three areas are commonly associated with emotional behaviours, and NPS is therefore implicated in stress-related behaviours. Indeed, forced swimming or restraint stress significantly activated NPS neurons in the pericoerulear region (peri-LC) and the Kölliker-Fuse nucleus of the lateral parabrachial area (KF-PBN) [24]. Intra-paraventricular nucleus (PVN) or *i.c.v.* administration of NPS in mice increased their locomotor and rearing activity, and plasma adrenocorticotropic hormone (ACTH) and corticosterone levels, suggesting that NPS can activate the arousal system and the hypothalamus-pituitary axis (HPA) [25].

The findings that both NPS and orexins are involved in the regulation of arousal, reward and pain suggest an interaction between the NPS [26] and orexin systems [9]. Indeed, it has been demonstrated that NPS (*i.c.v.*) can activate orexin neurons in the LH, PFA and DMH of rats [27, 28], where NPSRs are abundantly expressed [23]. Moreover, NPS has been reported to be an upstream activator of hypothalamic orexin neurons in feeding [27] and addiction [28, 29] behaviours. This suggests that NPS can activate orexin neurons and exert its biological functions, possibly including SIA, indirectly, by promoting the release of orexins.

Substance P is an undecapeptide belonging to the neurokinin (tachykinin) family [30] and exerts its effects mainly via NK_1_ receptors (NK_1_Rs) [31], a member of the GPCR family. Substance P is a well-known peripheral pronociceptive mediator [32] while it is antinociceptive at the supraspinal level [33]. In fact, intra-PAG microinjection (*i.pag.*) of substance P induces antinociception [34]. This effect may be mediated by the NK_1_Rs in the PAG since it is blocked by an NK_1_R antagonist and NK_1_Rs are densely distributed in pain-modulating brain regions including the PAG [35].

**Fig. 1:**
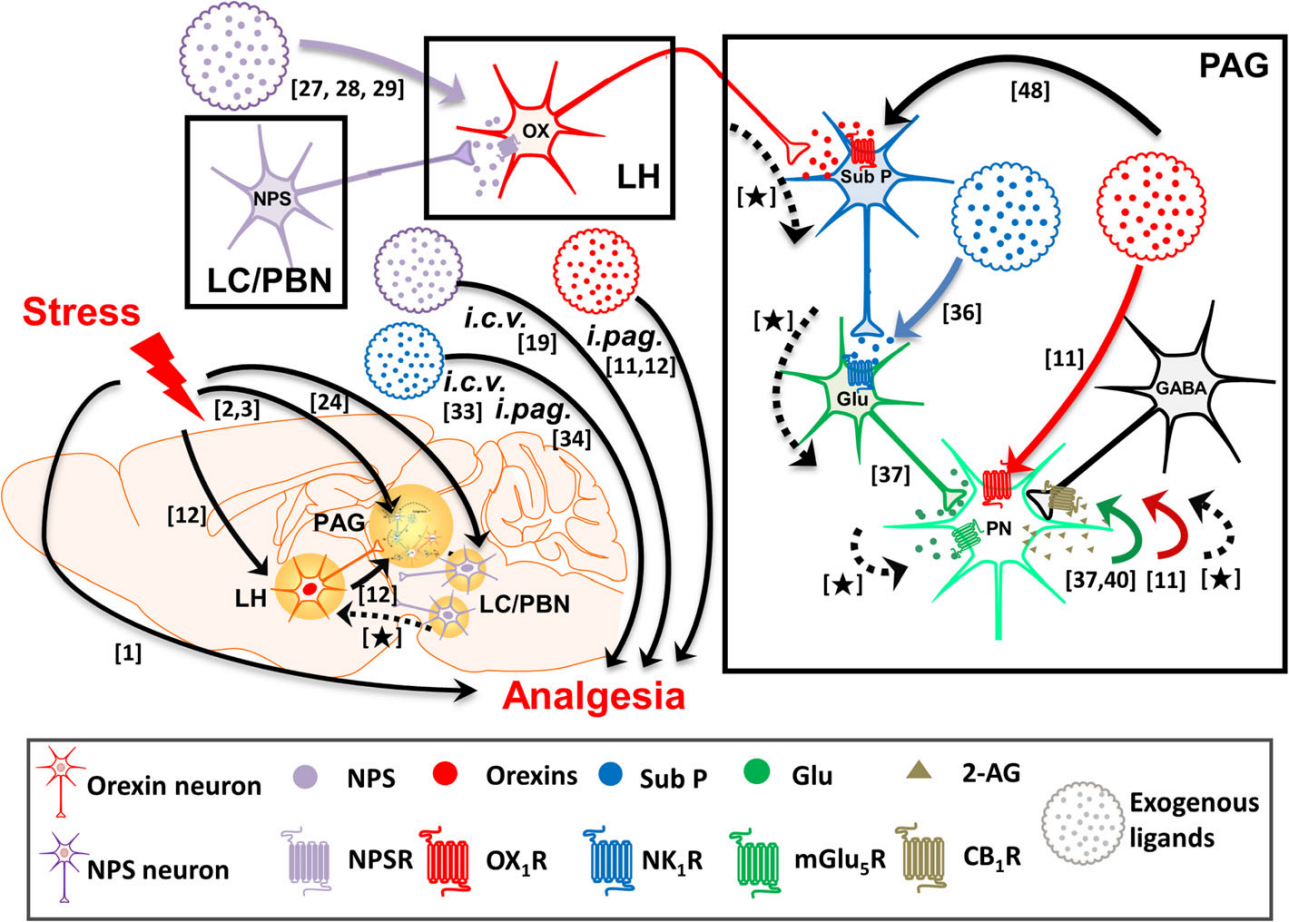
A schema depicting the possible relationships among NPS, orexins, substance P, mGlu_5_R and endocannabinoid (2-AG) during SIA, based on the available literature. The cascades occurring in the locus coeruleus (LC)/ parabrachial nucleus (PBN), lateral hypothalamus (LH) and periaqueductal gray (PAG) during stress or exposing to NPS (purple), orexins (red) or substance P (blue) are depicted in the right box. The findings that have been reported are shown by solid lines with the numbers of referred literatures. To fulfil our hypothesis, the links that have to be proven are now established in this study, which are denoted by broken lines marked with [★]. The images of mouse brain and neurons are adapted from Illustration Toolkit Neuroscience by Motifolio. PN: projection neuron; SubP: substance P; Glu: glutamate

Using an electrophysiological approach, Drew et al. (2009) [36] have investigated how substance P modulates synaptic transmission in brain slices containing the vlPAG. They demonstrated that substance P decreased evoked GABA release in vlPAG slices. This effect was abolished by an inhibitor of DAGL, a degradation enzyme of 2-AG, and an antagonist of mGlu_5_ receptors (mGlu_5_R). Importantly, a glutamate transporter inhibitor mimicked the GABA-reducing effect of substance P, but also occluded such action of substance P [37]. However, substance P markedly increased action potential-driven spontaneous glutamate release. It is suggested that substance P induces an enormous release of glutamate that may activate perisynaptic mGlu_5_R to initiate the eCB-mediated retrograde disinhibition mechanism in the vlPAG. They suggested this effect may contribute to the substance P-induced analgesic effect in the vlPAG [37], however no pain behaviors were not evaluated. Substance P in the PAG may also contribute to SIA since restraint stress [38] and LH stimulation [39] increased the substance P level in the PAG and *i.pag.* blockade of NK_1_Rs abolished LH-stimulation-induced antinociception. However, there are no direct in vivo studies supporting the involvement of PAG substance P in SIA. Taking into consideration the the complexity of the aforementioned neuropeptides in SIA, a scheme depicting the possible relationships among NPS, orexins, substance P, mGlu_5_R and eCB (2-AG) during SIA, based on the available literature, is illustrated in Fig. 1.

Substance P-induced vlPAG disinhibition, which is mediated through mGlu_5_R-initiated eCB signaling, highly resembles the OX_1_R-initiated 2-AG/CB_1_R signaling we reported previously [11], which contributes to SIA [12]. Moreover, mGlu_5_R [40] and eCBs [41, 42] are also involved in SIA. These events, all taking place in the vlPAG, prompted us to hypothesize the involvement of NK_1_Rs and mGlu_5_Rs in orexin-induced antinociception, and subsequently their involvement in SIA, possibly as downstream effectors of NPS. In this study, via behavioral, pharmacological and neurochemical approaches, we first examined the involvement of NK_1_R, mGlu_5_Rs and CB_1_Rs in substance P-induced antinociception. Next, we investigated whether orexins are upstream to substance P in the vlPAG in eliciting antinociceptive effects. Then, we examined whether NPS is an upstream modulator of LH orexin neurons. Lastly, we studied the involvement of the NPSR-OX_1_R-NK_1_R-mGlu_5_R-CB_1_R pathway in SIA.

## Materials and methods

### Animals

All animal experiments were approved by the Institutional Animal Care and Use Committee of College of Medicine, National Taiwan University following ARRIVE guidelines. Male C57BL/6 mice of 8–12 weeks were housed in groups of 10 in plastic cages and maintained in a holding room with a 12 h light-dark cycle with free access to food and water ad libitum. On the experimental day, mice were moved in their home cages to the behaviour room and acclimated there for at least 1 h before testing.

### Hot-plate test

The hot-plate test in mice was performed as reported previously [12]. Briefly, the mouse was placed on a hot-plate maintained at 50 °C and paw withdrawal latency was recorded with a cut-off time of 60 s to prevent tissue damage. The antinociceptive effect in each mouse at each time point was calculated as the % of the maximal possible effect (MPE) by the equation: %MPE = 100 x (Latency_after treatment_ - Latency_before treatment_) / (60s - Latency_before treatment_). The AUC of withdrawal latencies during the 60 min-recording period was calculated as the total antinociceptive effect in each mouse.

### SIA

To induce SIA, mice were restrained in a 50-ml centrifugal tube with several small holes for 30 min as reported previously [12]. The control non-stress group of mice remained at their home cages for the same 30 min before being subjected to the hot-plate test.

### Spontaneous locomotor activity

Locomotor activity was assessed by an open-field test in a 48 × 48 × 40 cm^3^ acrylic chamber with the arena floor divided into 36 squares, as described previously [43]. The mouse was placed in the center of the chamber, and the number of squares the mouse transpassed with forepaws (number of crossing) and the number of times the mouse stood up with forepaws on the floor (number of rearing) were counted for 5 min.

### Intra-vlPAG (*i.pag.*) and intra-cerebroventricular (*i.c.v.*) microinjection

When drugs were given by *i.pag.* or *i.c.v.* microinjection, mice received the *i.pag.* or *i.c.v.* cannulation surgery 1 week before the microinjection experiment, as reported previously [12]. Briefly, under anesthesia with 50 mg/kg Zoletil® 50 (a mixture of tiletamine and zolazepam) and xylazine (10 mg/kg), the mouse was placed in a stereotaxic apparatus and a 24-gauge, 10 mm stainless-steel guide cannula was implanted into the right vlPAG (− 4.8 mm caudal, − 0.5 mm lateral, − 2.8 mm ventral from bregma, Additional file 1: Figure S1A) or the right ventricle (− 0.5 mm caudal, − 1.0 mm lateral, − 2.2 mm ventral from bregma, Additional file 1: Figure S1B), according to stereotaxic coordinates of mice [44]. On the day of experiments, *i.pag.* or *i.c.v.* microinjection was performed through a 30-gauge injection needle (10 mm) connected to a Hamilton syringe (1.0 μl) on a microinfusion pump (KDS311, KD Scientific Inc., Holliston, MA, USA). The drug solution (0.1 μl) was delivered in 60 s, followed by a 240 s-residual time to avoid back-flow of drug solution. Nociceptive responses were measured 5 mins before as well as 5, 10, 20, 30 and 40 mins after *i.pag.* or *i.c.v.* microinjections. For the mice that underwent restraint stress, *i.pag* or *i.c.v.* microinjections of the antagonists were performed 5 mins before stress, and nociceptive responses were measured immediately, 5, 10, 20, 30 and 40 mins after stress. After the final behavioural evaluation, the animals were microinjected with 0.5 μl of 0.4% trypan blue solution (Sigma-Aldrich, St. Louis, MO, USA) through the guide cannula to verify the injection tract location. Animals were then sacrificed by decapitation, coronal brain sections (300 μm) were prepared on a vibratome (DSK microslicer DTK-1000, Dosaka, Japan). The injection site was identified by the presence of trypan blue stain diffusion in the vlPAG tissue. Only animals with the cannula correctly targeting the ventricle or vlPAG were included in data analysis.

### Measurements of substance P in vlPAG and NPS in LH of brain tissue homogenates

The preparation of the mouse vlPAG and LH homogenate is the same as previously reported [12]. Briefly, immediately after restraint stress, the mouse was sacrificed. Its brain was removed, placed in a pre-cooled adult mouse brain slicer matrix (Roboz Surgical Instrument, Gaithersburg, MD, USA), and sliced into 1 mm-thick coronal sections. vlPAG or LH brain tissues were bilaterally punched out with a 0.5 mm-tip according to a mouse brain atlas [44]. Each vlPAG sample was collected from one mouse brain, whereas each LH sample was from two mouse brains. After ultrasonication in lysis buffer, the lysates were homogenized and centrifuged (1900 g,14,000 rpm, 15 min) and supernatants collected. The protein concentration in the supernatant was measured by the Bradford method [45].

The substance P level in the vlPAG homogenate was measured with an EIA kit (Cat. No. 583751, Cayman Chemical. Ann Arbor, MI, USA) with a detection range of 3.9–500 pg/ml. The NPS level in the LH homogenates was measured with an ELISA kit. (Cat. No. CSB-EL016026MO, Cusabio, College Park, MD, USA) with a detection range of 4.69–300 pg/ml.

### Chemicals

NPS and [tBu-D-Gly^5^] NPS were synthesised and purified as previously described [46]. N-(2-methyl-6-benzoxazolyl)-N-1,5-naphthyridin-4-yl-urea (SB-334867, a selective OX_1_R antagonist), 6-methyl-2-(phenylethynyl) pyridine hydrochloride (MPEP, a selective mGlu_5_R antagonist) and orexin-A were purchased from Tocris Bioscience (Bristol, UK). Substance P, 1-(2,4-dichlorophenyl)-5-(4-iodophenyl)-4-methyl-N-1-piperidinyl-1H-pyrazole-3-carboxamide (AM251, a CB_1_R antagonist), and *cis*-2-(Diphenylmethyl)-N-[(2-iodophenyl)methyl]-1-azabicyclo [2.2.2] octan-3-amine oxalate salt (L-703,606, a selective NK_1_R antagonist) were purchased from Sigma-Aldrich. NPS and [tBu-D-Gly^5^] NPS were dissolved in 0.9% normal saline. Substance P was dissolved in 0.1 M acetic acid. SB-334867, L-703,606, MPEP and AM251 were dissolved in dimethyl sulfoxide (DMSO). All drugs were prepared at the working concentration for the intended *i.pag.* or *i.c.v.* injection doses.

### Statistical analysis

Data are expressed as the mean ± S.E.M. and the “n” indicates the number of mice tested in each group. In the hotplate test, two-way ANOVA with post hoc Bonferroni test was used to analyse time courses of antinociceptive effects among different groups. The antinociceptive effect was also assessed by the area under the curve (AUC) of the time courses of quantification of the line graph from baseline to the last time-point of the experiment. Each AUC bar graph was calculated by one-way ANOVA followed by Tukey’s multiple comparison test. Student’s T-test was employed to analyse the results obtained in EIA and ELISA tests. Differences were considered significant if *p* < 0.05.

## Results

### NPS is antinociceptive when given by *i.c.v.* but not *i.pag*. microinjection in mice

**Fig. 2:**
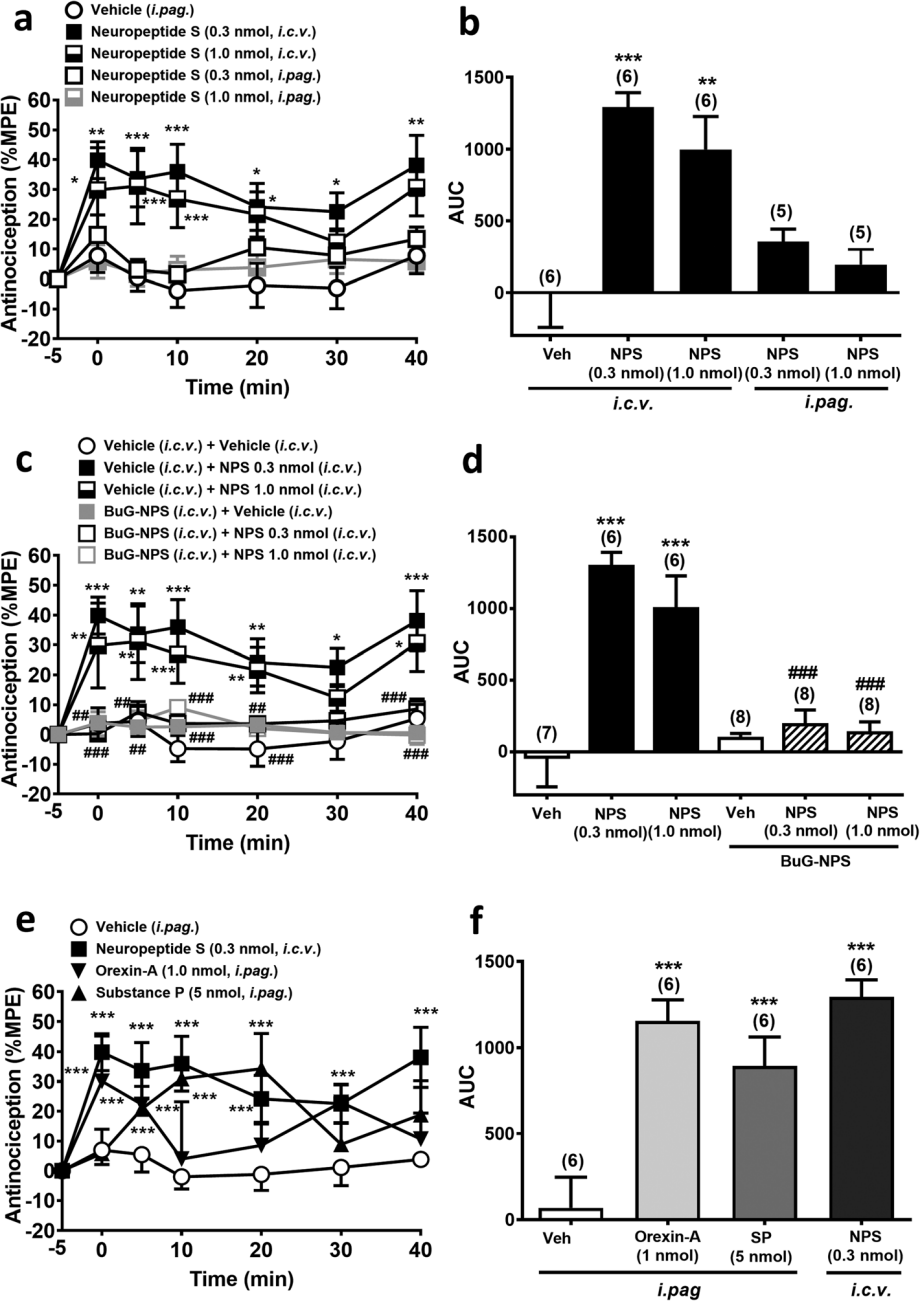
Antinociceptive effects induced by NPS, orexin-A and substance P in the mouse hot-plate test. **a-b**: Antinociceptive effects of NPS (0.3 & 1.0 nmol) by *i.c.v.* or *i.pag.* microinjection. **c-d**: Antinociceptive effects of *i.c.v.* NPS challenged by an NPSR antagonist, [tBu-D-Gly^5^] NPS (10 nmol, *i.c.v.*). **e-f:** A comparison of antinociceptive effects of orexin-A (1 nmol, *i.pag.*), substance P (5 nmol, *i.pag.*), and NPS (0.3 nmol, *i.c.v.*). **a, c** and **e**: The time course of the antinociceptive effect expressed as the percentage of the maximal possible effect (MPE) (two-way ANOVA /post hoc Bonferroni test). **b, d** and **f**: The area under the curve (AUC) of the % MPE measured within 40 min in each treatment group (one-way ANOVA /post hoc Tukey test**)**. The number denoted in the parentheses above each bar is the *n* number of mice tested in each group. Data are mean ± S.E.M. *p < 0.05, **p < 0.01, ***p < 0.001 vs. the vehicle control group, ^###^*p* < 0.001 vs. NPS 0.3 or 1.0 group

NPS when given by *i.c.v.* injection at the doses 0.3 and 1.0 nmol, which did not affect the spontaneous locomotor activity of mice (Additional file 2: Figure S2), significantly prolonged the latency of nociceptive response in the hot-plate test (Fig. 2a and b) in a time- (F_6,38_ = 5.696, *p* < 0.001, two-way ANOVA, Fig. 2a) and treatment-dependent (F_4,23_ = 10.25, *p* < 0.001, two-way ANOVA, Fig. 2a) manner. However, when NPS was given by *i.pag.* microinjection, it did not produce significant antinociceptive effect at either 0.3 or 1.0 nmol (Fig. 2a and b). This suggests that the site of action for NPS-induced supraspinal antinociception is brain region(s) other than the vlPAG.

### NPS (*i.c.v.*)-induced antinociception was antagonized by *i.c.v.* blockade of NPSRs

To investigate if the central antinociceptive effect of NPS is mediated via NPSR, we co-administered [tBu-D-Gly^5^] NPS (10 nmol, *i.c.v.*), a selective and potent NPSR antagonist [47], along with NPS (0.3 or 1.0 nmol, *i.c.v.*) to mice before the hot-plate test. [tBu-D-Gly^5^] NPS at 10 nmol (*i.c.v.*) did not affect the nociceptive response in naïve mice, but completely blocked the antinociceptive effect of *i.c.v.* NPS at the doses of 0.3 and 1.0 nmol (Fig. 2c and d) The overall comparison of the time course of the antinociceptive effect showed a significant difference between time and treatment (F_30,222_ = 1.872, *p* = 0.0057, two-way ANOVA, Fig. 2c). This suggests the central antinociceptive effect of NPS is mediated by NPSRs in the brain.

### NPS (0.3 nmol, *i.c.v.*), substance P (5 nmol, *i.pag.*) and orexin-A (1 nmol, *i.pag.*) induced comparable antinociceptive effects in mice

To substantiate our hypothesis that a cascade mediated by NPS, orexins and substance P sequentially is involved in SIA, we evaluated equipotent doses of these three neuropeptides in a concurrent behavioural assay. As shown in Fig. 2e and f, NPS (0.3 nmol, *i.c.v.*) produced a antinociceptive effect in the mouse hot-plate test that was comparable to the effects induced by *i.pag.* microinjection of orexin-A at 1 nmol and *i.pag.* substance P at 5 nmol, respectively, with a significant difference between time and treatment (F_18,120_ = 1.924, *p* = 0.0198, two-way ANOVA, Fig. 2e).

### Substance P (*i.pag*)-induced antinociception was antagonized by *i.pag.* blockade of NK_1_Rs, mGlu_5_Rs or CB_1_Rs

**Fig. 3:**
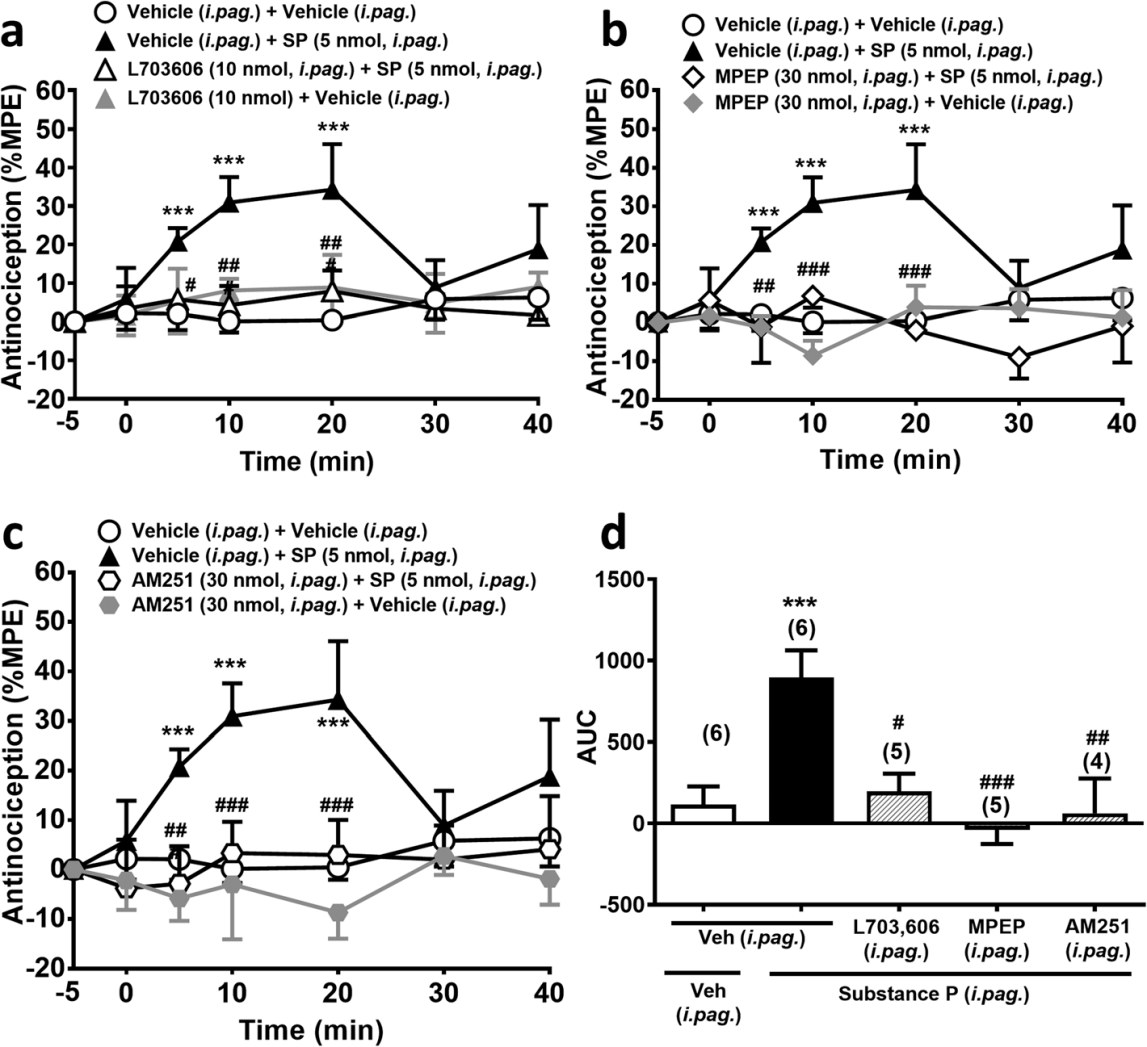
Substance P (*i.pag.*)-induced antinociception is antagonized by *i.pag.* blockade of NK_1_Rs, mGlu_5_Rs or CB_1_Rs. **a**-**c**: Time courses of antinociceptive effects (expressed as % MPE) induced by substance P (5 nmol, *i.pag.*) in combination with the vehicle or the antagonist of NK_1_Rs (L-703,606, 10 nmol, *i.pag.*, **a**, mGlu_5_Rs (MPEP, 30 nmol, *i.pag.*, **b**, and CB_1_Rs (AM251, 30 nmol, *i.pag.*, **c** in the mouse hot-plate test. (two-way ANOVA /post hoc Bonferroni test**). d:** The AUC of the antinociceptive effect in each treatment group **(**one-way ANOVA /post hoc Tukey test**)**. The antagonist was *i.pag.* co-administered with *i.pag.* substance P. The data presentation and statistics are the same as in Fig. 2. *p < 0.05, **p < 0.01, ***p < 0.001 vs. the vehicle control group; ^#^*p* < 0.05, ^##^*p* < 0.01, ^###^*p* < 0.001 vs. the Substance P group

To ascertain if the NK_1_R-mGlu_5_R-CB_1_R pathway revealed by the electrophysiological study of Drew et al. (2009) [36] is involved in *i.pag.* substance P-induced antinociceptive effect, we challenged the antinociceptive effect of substance P with selective antagonists of NK_1_Rs (L-703,606), mGlu_5_Rs (MPEP) and CB_1_Rs (AM251), respectively, in a treatment-dependent manner (F_3,18_ = 5.316, *p* = 0.0084, two-way ANOVA, Fig. 3a; F_3,18_ = 10.97, *p* = 0.0003, two-way ANOVA, Fig. 3b; F_3,17_ = 5.929, *p* = 0.0059, two-way ANOVA, Fig. 3c). Indeed, *i.pag.* co-administration of L-703,606 (10 nmol), MPEP (30 nmol) or AM251 (30 nmol) with substance P (5 nmol) significantly antagonized the antinociceptive effect of *i.pag.* substance P (Fig. 3).

### Orexin-A (*i.pag.*)-induced antinociception was antagonized by *i.pag.* blockade of NK_1_Rs and mGlu_5_Rs

**Fig. 4:**
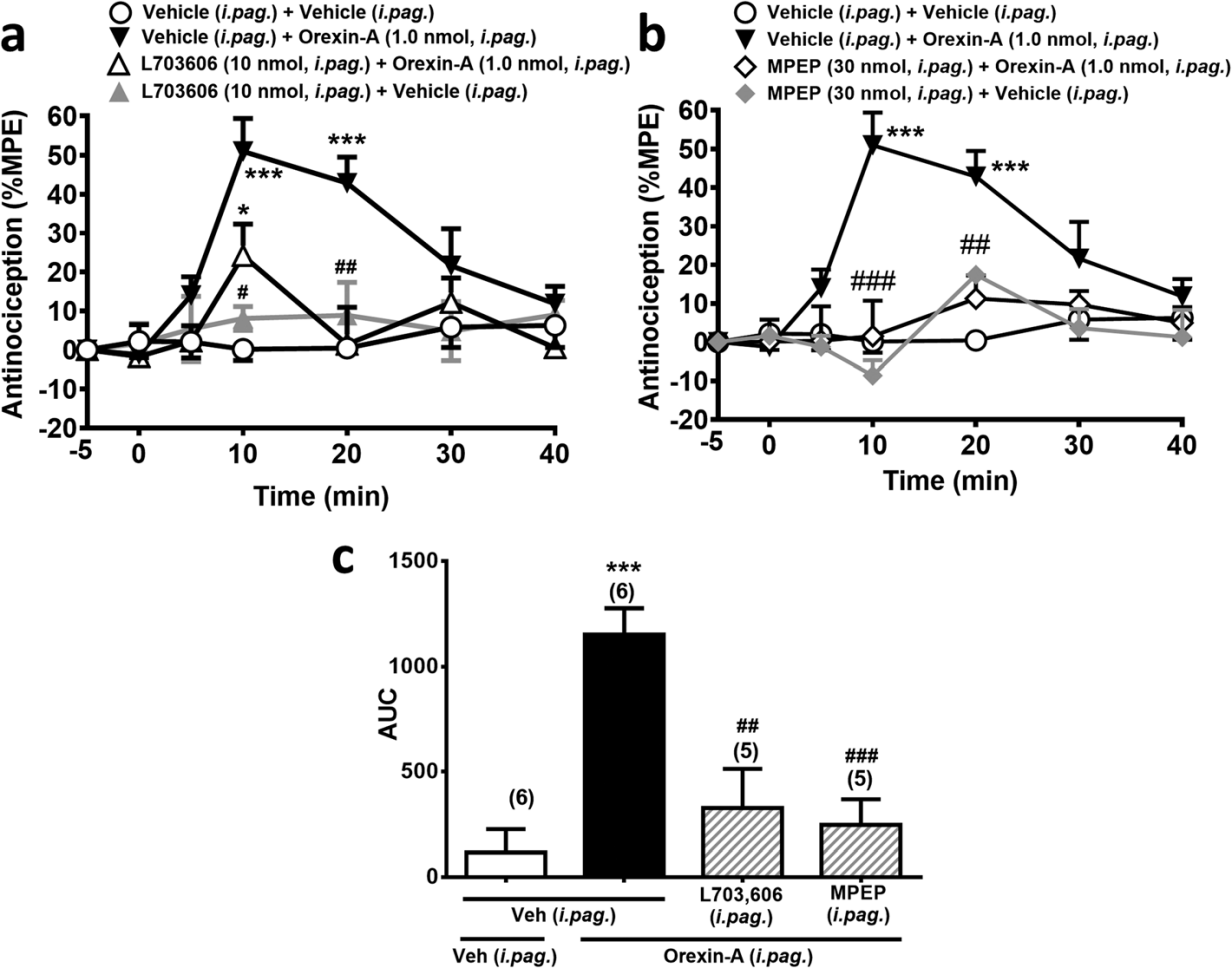
Orexin-A (*i.pag.*)-induced antinociception is antagonized by *i.pag.* blockade of NK_1_Rs or mGlu_5_Rs. **a**-**b**: Time courses of antinociceptive effects (expressed as % MPE) induced by orexin-A (1.0 nmol, *i.pag.*) in combination with the vehicle or the antagonist of NK_1_Rs (L-703,606, 10 nmol, *i.pag.,* **a** or mGlu_5_Rs (MPEP, 30 nmol, *i.pag.*, **b** in the mouse hot-plate test. (two-way ANOVA /post hoc Bonferroni test**). c:** The AUC of the antinociceptive effect in each treatment group **(**one-way ANOVA /post hoc Tukey test**)**. The antagonist was *i.pag.* co-administered with *i.pag.* orexin-A. The data presentation and statistics are the same as in Fig. 2. *p < 0.05, **p < 0.01, ***p < 0.001 vs. the vehicle control group; ^#^*p* < 0.05, ^##^*p* < 0.01, ^###^*p* < 0.001 vs. the orexin-A group

Our previous findings that *i.pag.* orexin-A induced antinociception through an OX_1_R-initiated eCB signalling [11], where the downstream mechanism was highly similar to *i.pag.* substance P induced antinociception as shown in the subsection above. In order to ascertain the interaction between orexin-A and substance P in the vlPAG, we challenged orexin-A-induced antinociception with *i.pag.* NK_1_R and mGlu_5_R antagonists, respectively. Co-administration of L-703,606 (10 nmol, *i.pag.*) or MPEP (30 nmol, *i.pag.*) significantly antagonized *i.pag*. orexin-A (1 nmol)-induced antinociception (Fig. 4). The overall comparison of time courses of the antinociceptive effect showed a significant difference between time and treatment (F_18,108_ = 3.841, *p* < 0.001, two-way ANOVA, Fig. 4a; F_18,108_ = 4.597, *p* < 0.001, two-way ANOVA, Fig. 4b). These results in combination with our previous findings suggest that orexin-A-induced antinociception is mediated by OX_1_Rs, NK_1_Rs, mGlu_5_Rs and CB_1_Rs sequentially in the vlPAG.

### NPS (*i.c.v.*)-induced antinociception was antagonized by *i.pag.* blockade of OX_1_Rs, NK_1_Rs, mGlu5Rs or CB_1_Rs

**Fig. 5:**
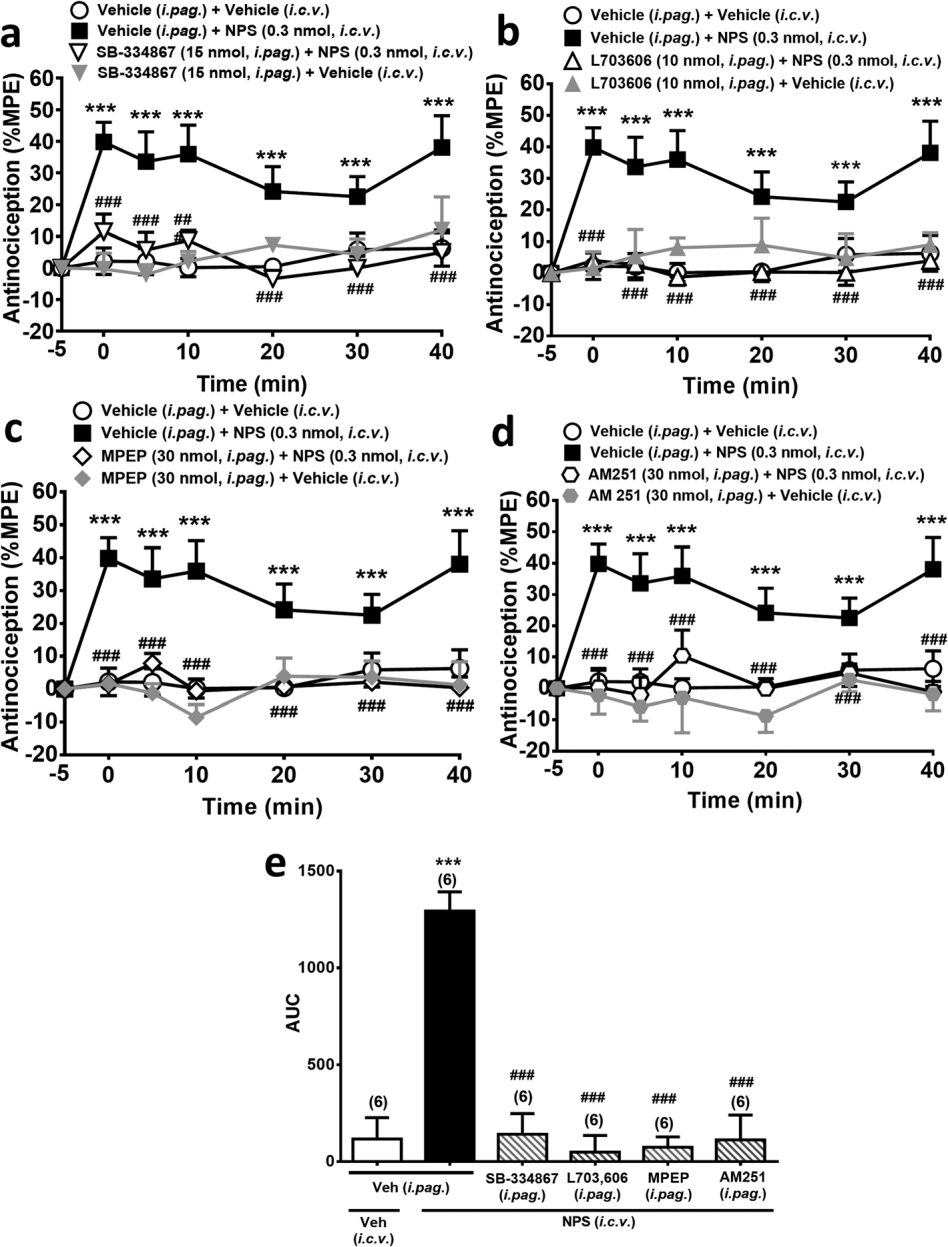
NPS (*i.c.v.*)-induced antinociception is antagonized by *i.pag.* blockade of OX_1_Rs, NK_1_Rs, mGlu_5_Rs or CB_1_Rs. **a**-**d**: Time courses of antinociceptive effects (expressed as % MPE) induced by NPS (0.3 nmol, *i.c.v.*) in combination with the vehicle or the antagonists of OX_1_Rs (SB-334867, 15 nmol, *i.pag.*), NK_1_Rs (L-703,606, 10 nmol, *i.pag.*), mGlu_5_Rs (MPEP, 30 nmol, *i.pag.*) or CB_1_Rs (AM251, 30 nmol, *i.pag.*) in the mouse hot-plate test. (two-way ANOVA /post hoc Bonferroni test**). e:** The AUC of the antinociceptive effect in each treatment group **(**one-way ANOVA /post hoc Tukey test**)**. The antagonist was *i.pag.* Administered immediately before *i.c.v.* injection of NPS. The data presentation and statistics are the same as in Fig. 2. *p < 0.05, **p < 0.01, ***p < 0.001 vs. the vehicle control group; ^#^*p* < 0.05, ^##^*p* < 0.01, ^###^*p* < 0.001 vs. the NPS group

Next, we investigated whether the now established OX_1_R-NK_1_R-mGlu_5_R-CB_1_R cascade in the vlPAG is involved in the supraspinal antinociceptive effect of NPS. Co-administration of the respective antagonists of OX_1_Rs (SB-334867, 15 nmol, *i.pag.*), NK_1_Rs (L-703,606, 10 nmol, *i.pag.*), mGlu_5_Rs (MPEP, 30 nmol, *i.pag.*) or CB_1_Rs (AM251, 30 nmol, *i.pag.*), significantly supressed the antinociceptive effect of *i.c.v.* NPS (0.3 nmol) (Fig. 5), in a time- (F_6,114_ = 3.252, *p* = 0.0055, two-way ANOVA, Fig. 5a; F_6,114_ = 2.936, *p* = 0.0106, two-way ANOVA, Fig. 5b; F_6,114_ = 2.603, *p* = 0.211, two-way ANOVA, Fig. 5c; F_6,114_ = 2.2, *p* = 0.0479, two-way ANOVA, Fig. 5d) and treatment- (F_3,19_ = 36.96, *p* < 0.001, two-way ANOVA, Fig. 5a; F_3,19_ = 28.58, *p* < 0.001, two-way ANOVA, Fig. 5b; F_3,19_ = 67.33, *p* < 0.001, two-way ANOVA, Fig. 5c; F_3,19_ = 23.44, *p* < 0.001, two-way ANOVA, Fig. 5d) dependent manner. Thus, *i.c.v.* NPS-induced analgesia is mediated by the OX_1_Rs, NK_1_Rs, mGlu_5_Rs and CB_1_Rs in the vlPAG.

### Restraint stress-induced analgesia was prevented by *i.c.v.* blockade of NPSR or *i.pag.* blockade of OX_1_Rs, NK_1_Rs or mGlu_5_Rs

**Fig. 6:**
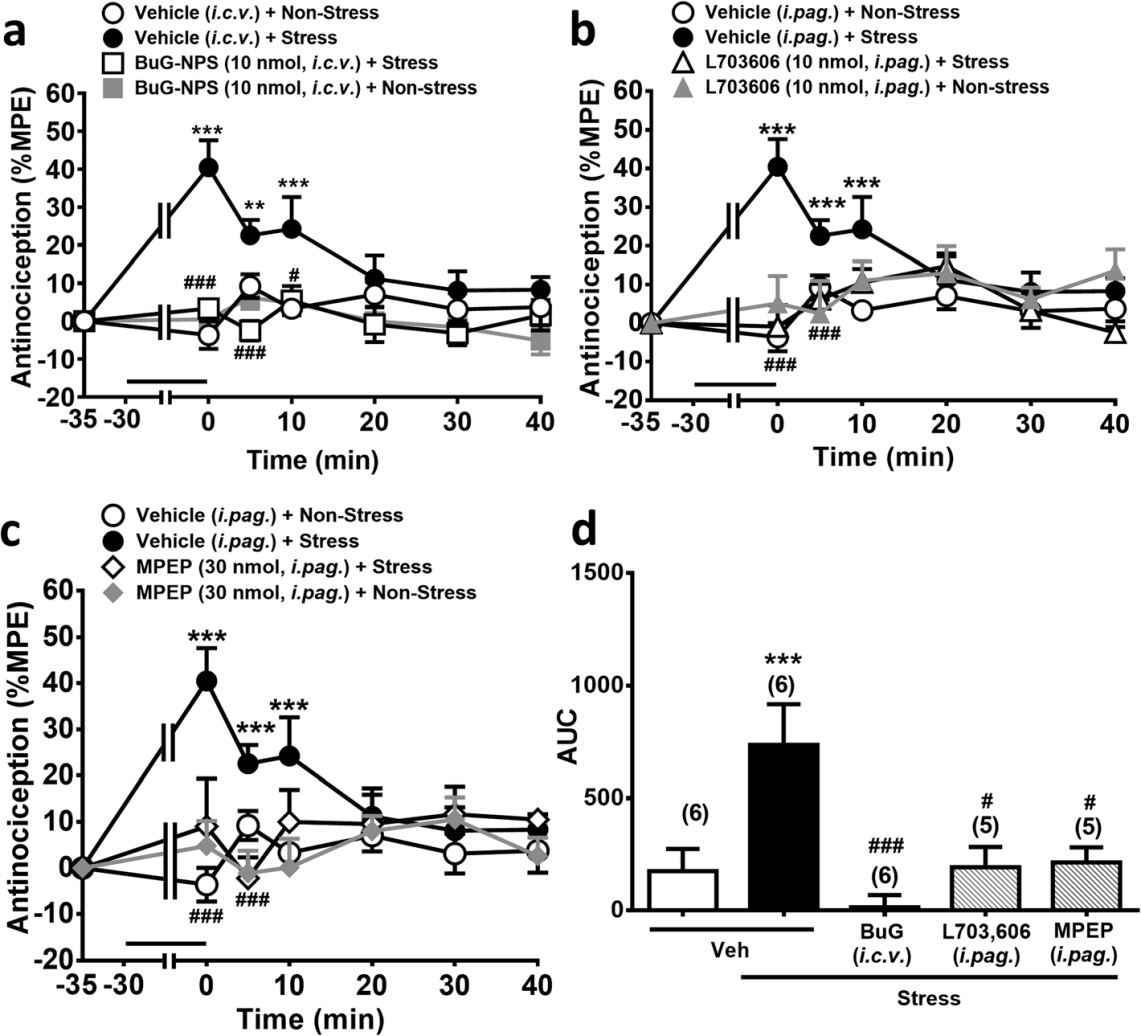
Restraint stress-induced antinociception (SIA) is prevented by *i.c.v.* blockade of NPSR or by *i.pag.* blockade of NK_1_Rs or mGlu5Rs. **a**-**c**: Time courses of antinociceptive effects (expressed as % MPE) induced by a 30 min-restraint stress (horizontal bars) in mice pre-treated with vehicle or the antagonist of NPSRs ([tBu-D-Gly^5^] NPS, 10 nmol, *i.c.v.*), NK_1_Rs (L-703,606, 10 nmol, *i.pag.*) or mGlu_5_Rs (MPEP, 30 nmol, *i.pag.*) in the hot-plate test. (two-way ANOVA /post hoc Bonferroni test). **d:** The AUC of the antinociceptive effect in each treatment group **(**one-way ANOVA /post hoc Tukey test**).** The antagonist was *i.c.v.* or *i.pag.* administered immediately before restraint stress. The data presentation and statistics are the same as in Fig. 2. *p < 0.05, **p < 0.01, ***p < 0.001 vs. the vehicle control group; ^#^*p* < 0.05, ^##^*p* < 0.01, ^###^*p* < 0.001 vs. the Stress group

We have previously demonstrated that the vlPAG OX_1_R-CB_1_R pathway is involved in the SIA induced by an acute restraint stress in mice [12]. We subsequently examined whether the now established NPSR-OX_1_R-NK_1_R-mGlu5R-CB_1_R cascade is involved in the SIA induced by the same restraint stress protocol. Mice receiving an acute restraint stress for 30 min exhibited significant reduced paw withdrawal response in the hot-plate test. This SIA diminished within 20 min (Fig. 6a) [12] and was significantly prevented in mice *i.c.v.* pre-treated with an NPSR antagonist ([tBu-D-Gly^5^] NPS, 10 nmol) (Fig. 6a and d) or with *i.pag.* Pretreated with NK_1_R (L-703,606, 10 nmol) (Fig. 6b and d) and mGlu_5_R (MPEP, 30 nmol) antagonists (Fig. 6c and d), respectively. The overall comparison of the time course of the antinociceptive effect showed a significant difference between time and treatment (F_18,114_ = 4.317, *p* < 0.001, two-way ANOVA, Fig. 6a; F_18,108_ = 3.780, *p* < 0.001, two-way ANOVA, Fig. 6b; F_18,108_ = 3.501, *p* < 0.001, two-way ANOVA, Fig. 6c). Take together with our previous findings that SB-334867 (15 nmol, *i.pag.*) and AM251 (30 nmol, *i.pag.*) prevented SIA [12], it is suggested that SIA is mediated via the NPSR-evoked OX_1_R-NK_1_R-mGlu_5_R-CB_1_R cascade in the vlPAG.

All the tested antagonists at the administered doses that attenuated SIA had no effect on the number of crossing and rearing, including the NPSR antagonist ([tBu-D-Gly^5^] NPS, 10 nmol, *i.c.v.*) (Additional file 3: Figure S3a and b), the NK_1_R antagonist (L-703,606, 10 nmol, *i.pag.*) (Additional file 3: Figure S3, C and D) and the mGlu_5_R antagonist (MPEP, 30 nmol, *i.pag.*) (Additional file 3: Figure S3**,** E and F), similar to the OX_1_R antagonist (SB-334867, 15 nmol, *i.pag.*) and the CB_1_R antagonist (AM251, 30 nmol, *i.pag.*) which we have reported previously [12]. This supports that these antagonists attenuate SIA by blocking their respective endogenous ligands.

### Restraint stress elevated NPS level in LH and substance P level in vlPAG

**Fig. 7:**
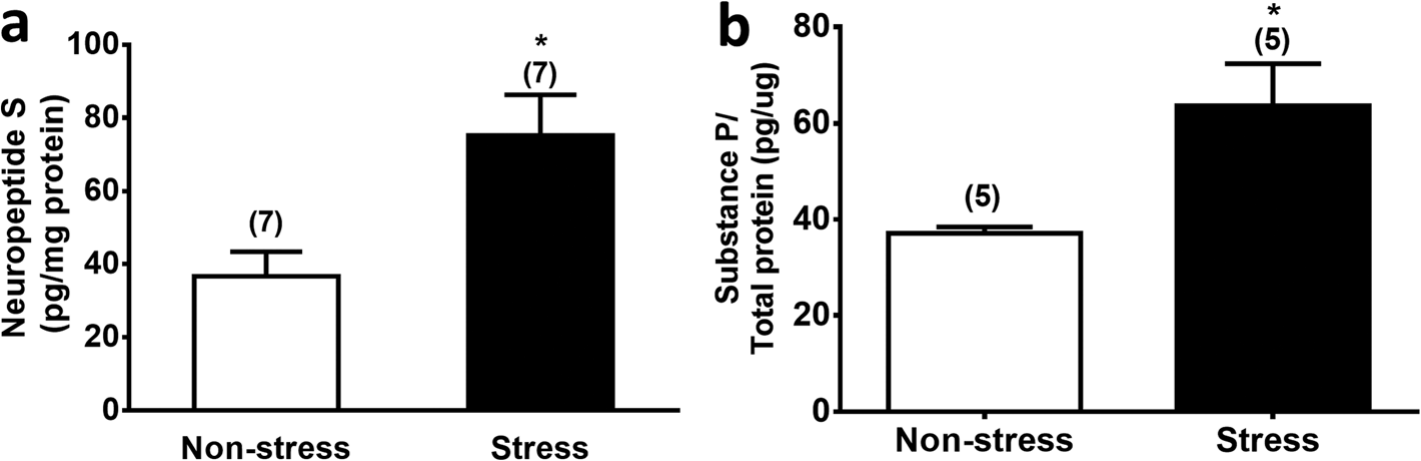
Restraint stress increases the NPS level in the LH (**a**) and the substance P level in the vlPAG. (**b**) Brain tissues containing the LH or vlPAG were punched and homogenized from restrained mice immediately after a 30 min-restraint stress (stress group) or from unrestrained control mice (non-stress group). NPS levels in LH homogenates were measured by an ELISA kit (Cusabio, College Park, MD, USA), whereas substance P level in vlPAG homogenates were measured by an EIA kit (Caymon Chemical. Ann Arbor, MI, USA). *p < 0.05, **p < 0.01 vs. the Non-stress control group (Student’s t-test)

Measurement of the neuropeptide content in the brain homogenate revealed that restrain stress significantly elevated the NPS level in the LH (df = 12, t = 2.987, *p* < 0.05, Student’s t-test, Fig. 7a) and the substance P level in the vlPAG (df = 9, t = 2.72, *p* < 0.05, Student’s t-test, Fig. 7b). Similar elevations in orexin-A levels were observed in the vlPAG of restrained mice as we previously reported [12].

## Discussion

**Fig. 8:**
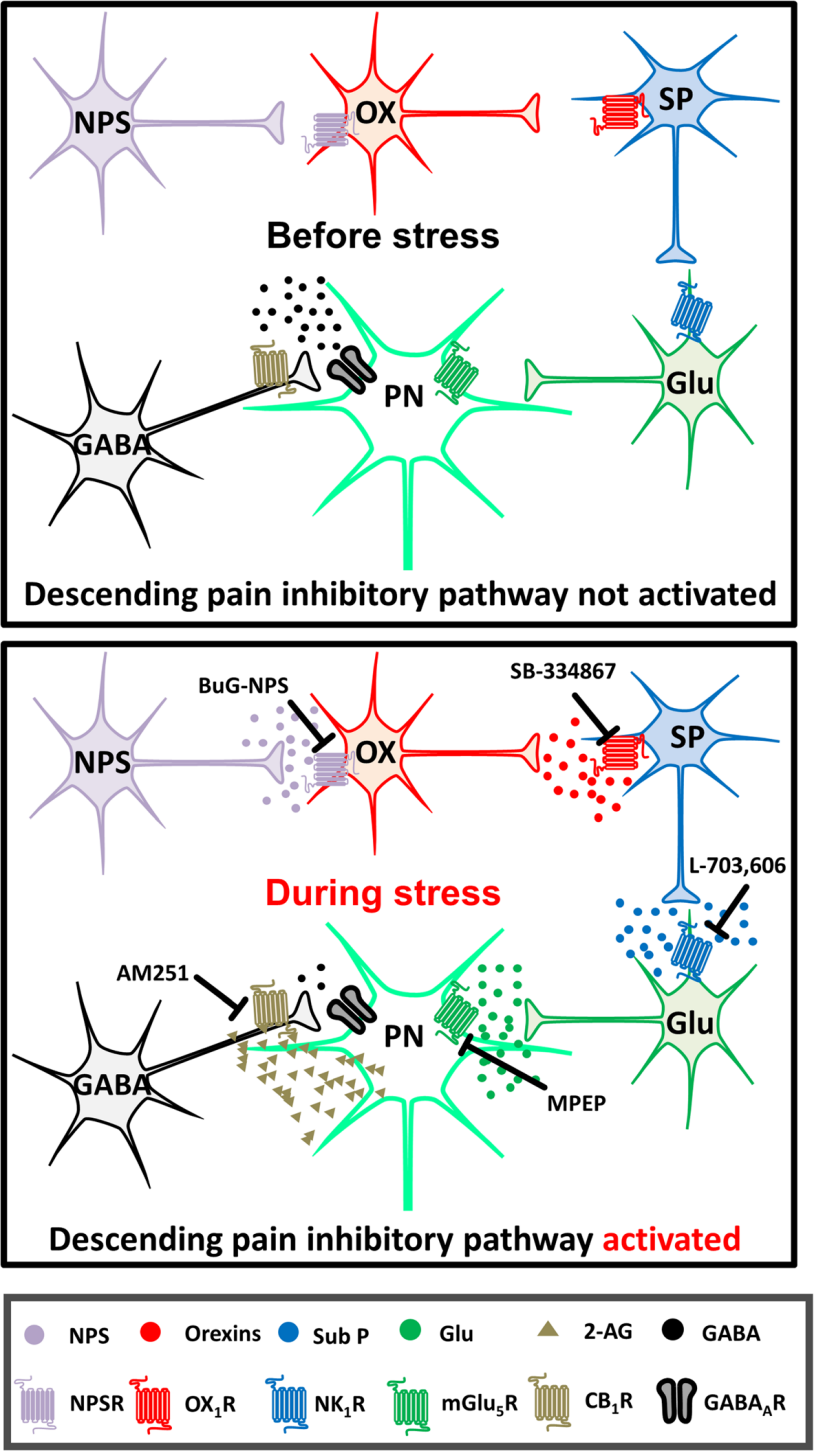
A proposed schema illustrating how NPS, orexins, substance P, mGlu_5_R and endocannabinoid (2-AG) may be involved in SIA. Before stress, the projection neurons in the vlPAG is under GABAergic inhibitory control. During stress, hypothalamic orexin neurons (OX) are activated by NPS, which is released possibly from the NPS neurosn in peri-LC and/or the KF-PBN in mice [24], releasing orexins that activate the OX_1_Rs on neurokinin (SubP) neurons and release substance P in the vlPAG. Then, substance P activates the NK_1_R-containing glutamate (Glu) neurons, yielding massive glutamate that in turn activates perisynaptic mGlu_5_Rs to initiate the GqPCR signalling and generate 2-AG. This endocannabinoid then retrogradely activates presynaptic CB_1_Rs to inhibit GABA release in the vlPAG, ultimately leading to analgesia. The points of pharmacological intervention performed in this study are marked with blunt arrows, labelled with the respective antagonists. The images of neurons are adapted from Illustration Toolkit Neuroscience by Motifolio. PN: projection neuron. GABA_A_R: GABA_A_ receptor

In this study, we found that the antinociceptive effect of *i.c.v.* NPS was blocked by *i.c.v.* injection of an NPSR antagonist and *i.pag.* injection of antagonists for OX_1_Rs, NK_1_Rs, mGlu_5_Rs and CB_1_Rs, respectively. These results suggest that orexins, substance P, glutamate and eCBs in the vlPAG are involved in supraspinal NPS-induced antinociception. In addition, blockade of either NPSRs, OX_1_Rs, NK_1_Rs, mGlu_5_Rs or CB_1_Rs suppressed the antinociception induced by a 30 min-restraint stress that increased the NPS level in the LH as well as the substance P level in the vlPAG. This suggests that NPS plays a role in SIA by activating the OX_1_R-NK_1_R-mGlu_5_R-CB_1_R-mediated sequential cascade that leads to antinociception through a disinhibition mechanism (i.e. inhibition of GABA release) mediated by GqPCR-PLC-DAGL-2-AG-CB_1_R signalling in the vlPAG [11, 12] (Fig. 8). Our results also suggest that restraint stress suppresses pain sensitivity in vivo by engaging the NPS-orexin-A-substance P-glutamate signalosome to initiate the eCB-mediated retrograde disinhibition mechanism in the vlPAG. Integrating with the existing literature, the findings from the present study may fill in the gaps, denoted as [★], among the signalling pathways of SIA as demonstrated by several research groups, as illustrated in Fig. 1.

### Substance P exerted antinociceptive effect via NK_1_Rs, mGlu_5_Rs and CB_1_Rs in the vlPAG

Drew et al. [36], using an electrophysiological approach, has shown that, in the vlPAG, substance P can facilitate the release of glutamate that subsequently activates postsynaptic mGlu_5_Rs located at perisynaptic sites, leading to the synthesis of 2-AG that retrogradely inhibits presynaptic GABA release via CB_1_Rs. Gregg et al. [40], using a behavioural approach, also demonstrated that activating mGlu_5_Rs in the PAG can induce an antinociceptive effect mediated by 2-AG and CB_1_Rs. Here, we further demonstrated that this substance P-initiated and mGlu_5_R-mediated eCB retrograde signalling contributes to the antinociceptive effect of substance P in the vlPAG since *i.pag.* substance P-induced antinociception was antagonized by *i.pag.* blockade of NK_1_Rs, mGlu_5_Rs or CB_1_Rs. The present study also supports that substance P is antinociceptive at the supraspinal level and the vlPAG is one of the sites of action.

### The substance P-NK_1_R-glutamate-mGlu_5_R cascade acts downstream of orexin-induced antinociception in the vlPAG

The finding that the NK_1_R antagonist attenuated orexin-induced antinociception in the vlPAG (Fig. 4) suggests that substance P acts downstream of orexin-induced antinociception. This finding is in agreement with a recent study that reported that the level of substance P in the vlPAG was increased following *i.pag.* orexin-A administration in rats [48]. Previously, we have shown that orexin via OX1Rs induces analgesia through a GqPCR-PLC-DAGL-2-AG-CB_1_R retrograde disinhibition mechanism in the vlPAG [11]. Given that mGlu_5_R, a GPCR, is also coupled to Gq proteins and mediates the antinociceptive effect via the same 2-AG-dependent disinhibition mechanism in the PAG [40] as the OX_1_R [11], it is reasonable to suggest that the mGlu_5_R is a downstream target after OX_1_R-NK_1_R activation. That is, orexin may induce analgesia via a cascade mediated by the OX_1_R-substance P-NK_1_R-glutamate-mGlu_5_R-PLC-DAGL-2-AG-CB_1_R signalling sequentially in the PAG (Fig. 8).

This sequential cascade may be able to explain the previous finding that *i.pag.* blockade of NK_1_Rs attenuated LH stimulation-induced antinociception [49]. It is likely that orexin is the mediator released from the LH to induce antinociception indirectly via the NK_1_R in the PAG. Additionally, involvement of substance P in the antinociceptive effect of orexin may also explain our previous electrophysiological finding that, in certain recorded vlPAG neurons, orexin-A did not induce postsynaptic depolarization but attenuated GABA release via presynaptic CB_1_Rs [11]. In addition to the 2-AG spill-over hypothesis, orexin-A may activate neurokinin neurons to release substance P that indirectly inhibits GABA release via mGlu_5_R-eCB signalling in those neurons that were not depolarized by orexin-A.

### The PAG is not the site of action for NPS-induced supraspinal antinociception

In agreement with previous studies that *i.c.v.* NPS was antinociceptive in swiss mice [19–21], we also found *i.c.v.* NPS reduced the hot-plate nociceptive response in C57BL/6JNarl mice. Peng et al. [20] suggested that the PAG is likely the site of action of NPS since *i.c.v.* NPS increased the c-Fos expression in the PAG where the NPSR mRNA is abundant [23]. However, our findings that direct *i.pag.* microinjection of NPS failed to induce antinociception and that *i.c.v.*, but not *i.pag.*, blockade of NPSRs antagonized *i.c.v.* NPS-induced antinociception indicate that *i.c.v.* NPS may act in brain regions other than the PAG to exert its antinociceptive effect.

### NPS-induced antinociception is mediated via OX_1_R-NK_1_R-mGlu_5_R-CB_1_R sequentially in the vlPAG

The findings that *i.pag.* blockade of OX_1_Rs, NK_1_Rs, mGlu_5_Rs and CB_1_Rs prevented *i.c.v.* NPS-induced antinociception suggest the involvement of the OX_1_R-NK_1_R-mGlu_5_R-CB_1_R signalling in the vlPAG in the supraspinal antinociceptive action of NPS. The site of action is likely in the hypothalamic areas where orexin neurons are located, especially the LH that is involved in pain regulation. Ideally, it would be more precise to study the action of NPS and its antagonist on orexin neurons in the LH via intra-LH microinjection. However, due to the difficulty of performing both intra-LH and *i.pag*. cannulations in mice, *i.c.v.* and *i.pag*. microinjections were employed (Figs. 5 and 6). Nevertheless, several studies have suggested an interaction between NPS and orexin systems. Anatomical and functional studies suggest that NPS can activate orexin neurons and may modulate biological functions indirectly via released orexins. First, the hypothalamic regions where orexin neurons are located, including the LH, PFA, and DMH, are enriched with NPSRs [23]. Second, after *i.c.v.* injection of NPS in rats, fos-immunoreactive cells in the hypothalamus, especially in the LH, were orexin-A-positive [27, 28]. Third, NPS has been reported to be an upstream activator of the orexin system in feeding [27] and addiction [28] behaviours acting in the hypothalamus. Therefore, it is likely that NPS activates the orexin neurons at the LH, releasing orexins in the vlPAG to induce antinociception.

### SIA is mediated by endogenous NPS-initiated hypothalamic orexins via the OX_1_R-NK_1_R-mGlu_5_R-CB_1_R-mediated sequential cascade in the vlPAG

Previously, we have demonstrated that SIA is mediated by orexins released from the LH, an important region for SIA [13], via an OX_1_R-initiated 2-AG-dependent disinhibition mechanism in the vlPAG [12]. Here, we extend the findings in this study to suggest that NPS activates hypothalamic orexin neurons and add substance P as downstream of vlPAG OX_1_R activation in this SIA mechanism. That is, during stress, hypothalamic orexin neurons are activated by NPS, which is released possibly from the peri-LC and/or the KF-PBN in mice [24], releasing orexins that activate the OX_1_Rs on neurokinin neurons in the vlPAG. Then, substance P is released and activates the NK_1_R-containing glutamate neurons, yielding massive glutamate release that in turn activates perisynaptic mGlu_5_Rs to initiate GqPCR signalling and generation of 2-AG. This eCB then retrogradely activates presynaptic CB_1_Rs to inhibit GABA release in the vlPAG, ultimately leading to analgesia (Figs. 1 and 8). This conclusion is based on the following findings, which may fill the gaps [★] in the schema depicted in Fig. 1, that (1) stress increased NPS levels in the LH (Fig. 7a) and SIA was reduced by blocking NPSRs (Fig. 6a); (2) stress increased orexin levels in the vlPAG and SIA was reduced by blocking OX_1_Rs in the vlPAG [12]; (3) stress increased substance P levels (Fig. 7b) and SIA was reduced by blocking NK_1_Rs in the vlPAG (Fig. 6b); (4) SIA was reduced by blocking either mGlu_5_Rs (Fig. 6c), CB_1_Rs or DAGL in the vlPAG [12]. The antagonist/inhibitor of NPSRs (BuG-NPS, Fig. 6a), OX_1_Rs (SB-334867) [12], NK_1_Rs (L-703,606, Fig. 6b), mGlu_5_Rs (MPEP, Fig. 6c), CB_1_Rs (AM251) or DAGL (tetrahydrolipstatin) [12] employed at the dose blocking SIA, per se, did not affect nociceptive threshold in unrestrained normal mice, suggesting no non-specific effects of these antagonists employed at the concentrations used in this study.

Since 1990s, substance P has been reported to play a role in SIA while the site(s) of action remain unidentified. Rosen et al. [38] reported that substance P was released from the PAG of animals in response to a behavioural stress, suggesting that endogenous substance P contributes to SIA originated from the PAG-mediated descending pain inhibition. The finding that the antinociceptive effect induced by stimulating the LH was abolished by *i.pag.* L-703,606 [39], suggesting that stimulating the LH can release substance P to induce antinociception via the NK_1_Rs in the PAG. Here, we provided direct evidence supporting that SIA is mediated by elevated substance P in the PAG.

Several lines of evidence have indicated the involvement of NPS in stress-induced responses. NPSRs are enriched in the amygdala and hypothalamus [22], stress-related brain regions. The number of c-fos-containing NPS neurons in the peri-LC and KF-PBN was increased after a short-term forced swim stress or restraint stress [24]. The current finding that the acute restraint stress that induces analgesia can increase the NPS level in the LH directly supports that NPS is released during stress and contributes to SIA.

Several reports have indicated a cross-modulatory relationship between NPS and the corticotrophin releasing factor (CRF) system in stress-related responses. Paneda et al. [50] reported that CRF_1_ receptor may mediate NPS-induced cocaine reinstatement in mice. Conversely, Jungling et al. [4]. demonstrated that CRF can modulate NPS neurons in the LC of mice following acute stress. It remains to be elucidated if interactions between the CRF system and the NPSR-OX_1_R-NK_1_R-mGlu_5_R-CB_1_R-mediated sequential cascade in SIA occur.

### Limitations of the current study

In the current study, we found that NPS (*i.c.v.*) at 0.3 and 1 nmol in C57BL/6 did not induce significant hyperlocomotion (Additional file 2: Figure S2). This is different with previous studies, where *i.c.v.* NPS at doses of 0.1 and 1.0 nmol induced hyperlocomotion in C57BL/6 [50] and Swiss mice [4]. However, Rizzi et al. [51], Castro et al. [52] and Boeck et al. [53] consistently demonstrated that *i.c.v.* NPS, only at the dose of 0.1 nmol, but not 0.01 and 1.0 nmol, exhibited significant hyperlocomotion in CF-1 mice. Furthemore, Holanda et al. [21] reported that *i.c.v.* NPS at 0.1 nmol did not increase locomotor activity in CF-1 mice. The discrepancy among studies is unclear. It may be that the *i.c.v.* NPS doses employed under the conditions (mouse strain and the motor activity assessment) in the present study fall outside the optimal dose for inducing hyperlocomotion.

## Conclusions

During stress, NPS is released to activate hypothalamic orexin neurons, releasing orexins that activate OX_1_Rs on neurokinin neurons in the vlPAG, releasing substance P that activates NK_1_Rs on glutamate neurons, yielding massive glutamate that in turn activates perisynaptic mGlu_5_Rs to initiate the G_q_PCR signalling and then generate 2-AG, which then. Retrogradely activates presynaptic CB_1_Rs to inhibit GABA release in the vlPAG, ultimately leading to analgesia (Fig. 8).

## Supplementary information

**Additional file 1:** **Figure S1.** The representative diagram of *i.pag.* (A) and *i.c.v.* (B) microinjections in mice. The diagrams were adapted from mouse brain atlas [44]. The black dots (**A**) and white dots (**B**) represent the microinjection sites.

**Additional file 2:** **Figure S2.** Effects of NPS on locomotor activity. Locomotor activity in the open field test was measured before and 10 min after *i.c.v.* administration of 0.3 nmol (**A-B**) or 1 nmol (**C-D**) of NPS. Locomotor activity was assessed by the number of crossing (**A & C**) and rearing (**B & D**) in the open field test for 5 min. Data are expressed as the mean ± S.E.M. (Unpaired t-test)

**Additional file 3:** **Figure S3.** Effects of [tBu-D-Gly^5^] NPS, L-703,606 or MPEP on locomotor activity. Locomotor activity in the open field test was measured before and 10 min after administration of [tBu-D-Gly^5^] NPS (10 nmol, *i.c.v.*) (**A-B**), L-703,606 (10 nmol, *i.pag.*) (**C-D**), or MPEP (30 nmol, *i.pag.*) (**E-F**). Locomotor activity was assessed by the number of crossing (**A, C & E**) and rearing (**B, D & F**) in the open field test for 5 min. Data are expressed as the mean ± S.E.M. (Unpaired t-test)

## Data Availability

All data generated or analyzed during this study are included in this published article and its supplementary information files.

## Abbreviations

2-AG: 2-arachidonoylglycerol
AM251: 1-(2,4-Dichlorophenyl)-5-(4-iodophenyl)-4-methyl-N-1-piperidinyl-1H-pyrazole-3-carboxamide,CB_1_R, CB_1_ receptor
CRF: Corticotrophin releasing factor
DAGL: Diacylglycerol lipase
DMH: Dorsomedial hypothalamus
eCB: endocannabinoid
EIA: Enzyme immunoassay
ELISA: Enzyme-linked immunosorbent assay
GqPCR: Gq-protein coupled receptor
HPA: Hypothalamus-pituitary axis
*i.c.v.*: intra-cerebroventricular
*i.pag*: intra-ventrolateral periaqueductal gray
L-703,606: cis-2-(Diphenylmethyl)-N-[(2-iodophenyl)methyl]-1-azabicyclo [2.2.2] octan-3-amine oxalate salt
LC: Locus coeruleus
LH: Lateral hypothalamus
mGlu_5_R: mGlu_5_ receptor
MPE: Maximal possible effect
MPEP: 2-methyl-6-(phenylethynyl) pyridine hydrochloride
NK_1_R: NK_1_ receptor
NPS: Neuropeptide S
NPSR: Neuropeptide S receptor
OX_1_R: OX_1_ receptor
OX_2_R: OX_2_ receptor
PBN: Parabrachial nucleus
PFA: Perifornical area
PLC: Phospholipase C
PVN: Paraventricular nucleus
SB-334867: N-(2-Methyl-6-benzoxazolyl)-N′-1,5-naphthyridin-4-yl urea
SIA: Stress-induced analgesia
vlPAG: ventrolateral periaqueductal gray
