# Supplementary material for: Neuropeptide S-initiated sequential cascade mediated by OX1, NK1, mGlu5 and CB1 receptors: a pivotal role in stress-induced analgesia
Source: J Biomed Sci. 2020 Jan 9;27:7. doi: 10.1186/s12929-019-0590-1 (PMC6950992; doi:10.1186/s12929-019-0590-1)
Supplement: Supplementary file 1 — Additional file 1: Figure S1. The representative diagram of i.pag. (A) and i.c.v. (B) microinjections in mice. The diagrams were adapted from mouse brain atlas [44]. The black dots (A) and white dots (B) represent the microinjection sites. [file 12929_2019_590_MOESM1_ESM.docx]

**Additional file 1**
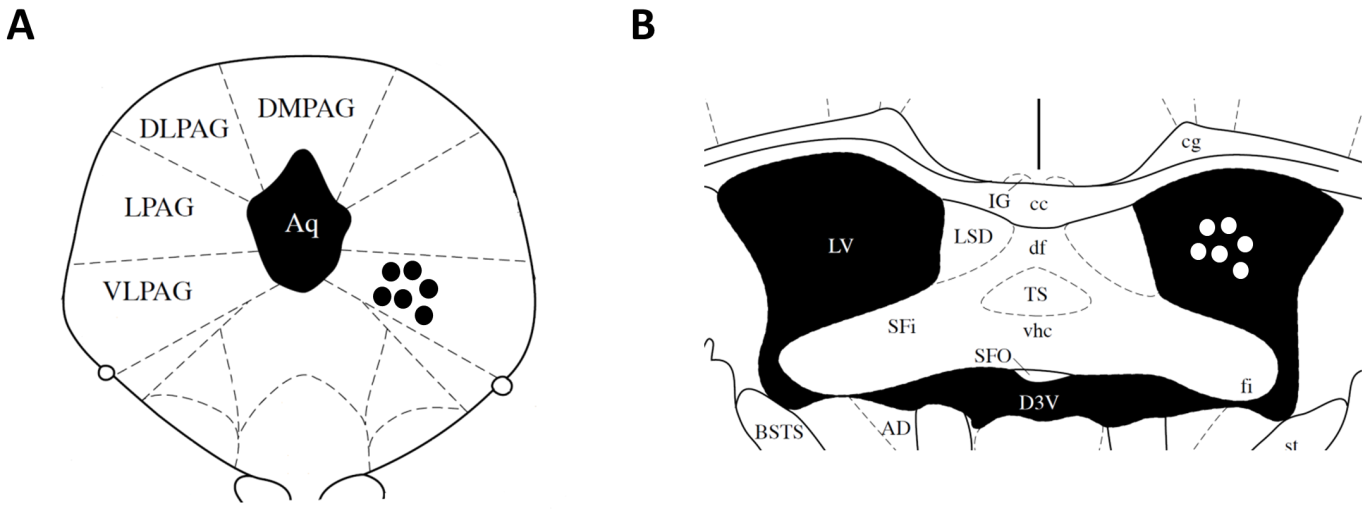


**Figure S1. The representative diagram of *i.pag.* (A) and *i.c.v.* (B) microinjections in mice.** The diagrams were adapted from mouse brain atlas [44]. The black dots (**A**) and white dots (**B**) represent the microinjection sites.
