# Supplementary material for: Neuropeptide S-initiated sequential cascade mediated by OX1, NK1, mGlu5 and CB1 receptors: a pivotal role in stress-induced analgesia
Source: J Biomed Sci. 2020 Jan 9;27:7. doi: 10.1186/s12929-019-0590-1 (PMC6950992; doi:10.1186/s12929-019-0590-1)
Supplement: Supplementary file 2 — Additional file 2: Figure S2. Effects of NPS on locomotor activity. Locomotor activity in the open field test was measured before and 10 min after i.c.v. administration of 0.3 nmol (A-B) or 1 nmol (C-D) of NPS. Locomotor activity was assessed by the number of crossing (A & C) and rearing (B & D) in the open field test for 5 min. Data are expressed as the mean ± S.E.M. (Unpaired t-test) [file 12929_2019_590_MOESM2_ESM.docx]

Additional file 2


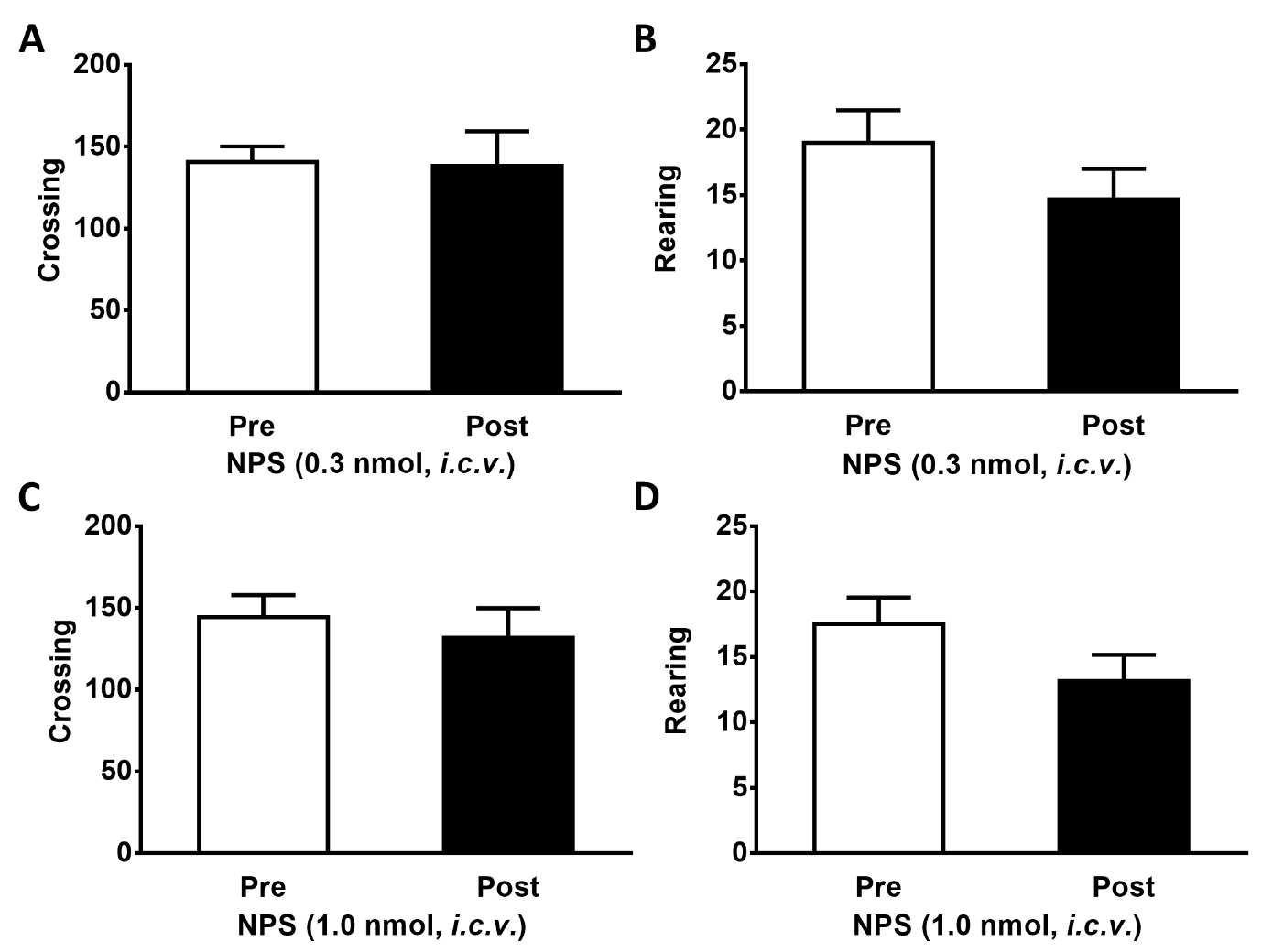


**Figure S2. Effects of NPS on locomotor activity**. Locomotor activity in the open field test was measured before and 10 min after *i.c.v.* administration of 0.3 nmol (**A-B**) or 1 nmol (**C-D**) of NPS. Locomotor activity was assessed by the number of crossing (**A & C**) and rearing (**B & D**) in the open field test for 5 min. Data are expressed as the mean ± S.E.M. (Unpaired t-test)
