# Supplementary material for: Neuropeptide S-initiated sequential cascade mediated by OX1, NK1, mGlu5 and CB1 receptors: a pivotal role in stress-induced analgesia
Source: J Biomed Sci. 2020 Jan 9;27:7. doi: 10.1186/s12929-019-0590-1 (PMC6950992; doi:10.1186/s12929-019-0590-1)
Supplement: Supplementary file 3 — Additional file 3: Figure S3. Effects of [tBu-D-Gly5] NPS, L-703,606 or MPEP on locomotor activity. Locomotor activity in the open field test was measured before and 10 min after administration of [tBu-D-Gly5] NPS (10 nmol, i.c.v.) (A-B), L-703,606 (10 nmol, i.pag.) (C-D), or MPEP (30 nmol, i.pag.) (E-F). Locomotor activity was assessed by the number of crossing (A, C & E) and rearing (B, D & F) in the open field test for 5 min. Data are expressed as the mean ± S.E.M. (Unpaired t-test) [file 12929_2019_590_MOESM3_ESM.docx]

**Additional file 3**


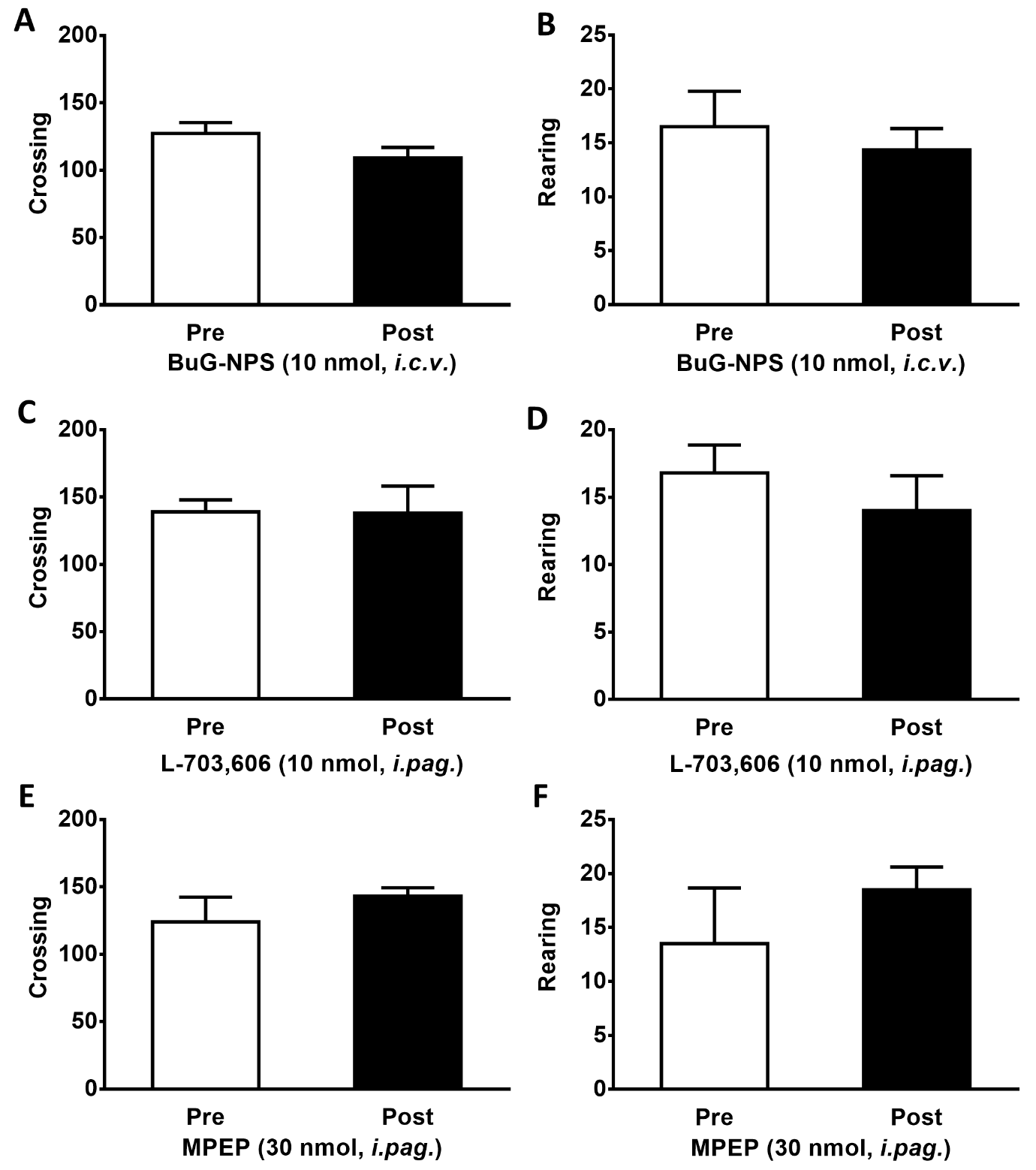


**Figure S3. Effects of [tBu-D-Gly^5^]NPS, L-703,606 or MPEP on locomotor activity**. Locomotor activity in the open field test was measured before and 10 min after administration of [tBu-D-Gly^5^]NPS (10 nmol, *i.c.v.*) (**A-B**), L-703,606 (10 nmol, *i.pag.*) (**C-D**), or MPEP (30 nmol, *i.pag.*) (**E-F**). Locomotor activity was assessed by the number of crossing (**A, C & E**) and rearing (**B, D & F**) in the open field test for 5 min. Data are expressed as the mean ± S.E.M. (Unpaired t-test)
